# Rapid Action of Retinoic Acid on the Hypothalamic Pituitary Adrenal Axis

**DOI:** 10.3389/fnmol.2019.00259

**Published:** 2019-10-30

**Authors:** Peter I. Imoesi, Ellen E. Bowman, Patrick N. Stoney, Sylwia Matz, Peter McCaffery

**Affiliations:** ^1^Institute of Medical Sciences, School of Medicine, Medical Sciences and Nutrition, University of Aberdeen, Aberdeen, United Kingdom; ^2^Cell Signal Unit, Okinawa Institute of Science and Technology, Okinawa, Japan

**Keywords:** retinoic acid, hypothalamic pituitary adrenal axis, corticosterone, aldosterone synthesis, adrenal gland, Cyp11b1, Cyp11b2

## Abstract

Retinoic acid (RA) is the active metabolite of vitamin A but is also used as a medication, primarily for acne in which the treatment regime lasts several months. A number of studies have indicated that treatment with RA over this time period impacts the hypothalamic-pituitary-adrenal (HPA) axis and may contribute to a number of the side-effects of the drug. No studies though have investigated the short-term, early effects RA may have on the HPA axis *via* the transcriptional pathways activated by the RA receptor. This study investigated the action of RA over 3 days on regulatory components of the HPA axis. Several key genes involved in glucocorticoid feedback pathways in the hippocampus, hypothalamus and pituitary were unchanged after 3-days exposure to RA. Key elements though in the adrenal gland involved in corticosterone and aldosterone synthesis were altered in particular with the *Cyp11b2* gene downregulated *in vivo* and *ex vivo*. The rapid, 5 h, change in *Cyp11b2* expression suggested this activation may be direct. These results highlight the adrenal gland as a target of short-term action of RA and potentially a trigger component in the mechanisms by which the long-term adverse effects of RA treatment occur.

## Introduction

Isotretinoin, the 13-*cis* isomer of retinoic acid (RA), is an effective oral treatment for severe acne first approved in the US, with FDA approval in 1982 (Layton, 2009). Its success resulted in wide usage and it is reported that by 2011 20 million patients had taken it worldwide (Lowenstein and Lowenstein, 2011). Early studies suggested that isotretinoin did not affect hormone levels (Palatsi et al., 1997), but subsequent investigation revealed that, to the contrary, its effects on hormone balance were broad with a complex array of effects over a typical 3-month treatment regime. Isotretinoin was reported to repress level of numerous hormones (Karadag et al., 2015), reducing adrenocorticotropic hormone (ACTH), cortisol, luteinizing hormone, prolactin, thyroid hormone and testosterone, as well as growth factors such as insulin-like growth factor-binding protein 3, insulin-like growth factor 1 (IGF-1) and growth hormone (GH). The effects were sufficiently strong for isotretinoin to be considered a potential treatment for Cushing’s syndrome (Vilar et al., 2016).

Given these wide-ranging hormonal effects, it is perhaps unsurprising that up to 90% of patients taking isotretinoin reported at least one side effect (Bremner et al., 2012). Depression has been one adverse effect associated with isotretinoin treatment (Bremner and McCaffery, 2008), a side-effect contentious ever since the drug was first marketed and for which controversy still continues (Oliveira et al., 2018). Research to determine a plausible cellular and molecular mechanism has pointed to the contribution of reduced hippocampal neurogenesis (Crandall et al., 2004) and, more recently, induction of the hypothalamic-pituitary-adrenal (HPA) axis given the link between HPA axis hyperactivity and depression (Pariante and Lightman, 2008). Several studies on the chronic effects of RA have suggested that this drug can induce hyperactivity of the HPA axis (Cai et al., 2010). Long-term exposure to RA results in significantly increased expression of corticotrophin-releasing hormone receptor 1 (CRHR1), estrogen receptor-β (ERβ) and mineralocorticoid receptor (MR) in the hypothalamus, three genes involved in the activation of corticotrophin-releasing hormone (CRH) neurons while the ratio of GRα/MR declines (Cai et al., 2015).

To date the focus of research has been on the chronic effects of RA on the body, acting over several weeks or months (Karadag et al., 2015). No study has examined the short-term effects of RA as a regulator of gene expression; the primary action of RA is *via* retinoic acid receptors (RARs) which are ligand-activated transcription factors. The present study investigated the effect of RA over 3 days on the HPA axis of the adult rat and found that early effects on the HPA axis in the adult were primarily directed to the adrenal gland.

## Materials and Methods

### Animals

The *in vivo* studies used 8-week-old, Sprague–Dawley male rats (Charles River) which were randomly divided into a treatment group (*n* = 5) and a control group (*n* = 5). For the *ex vivo* experiments, adrenal glands were taken from 14 male Sprague–Dawley pups (9–10 days old), which were euthanized by a lethal intraperitoneal injection of pentobarbital. The pups were decapitated before the adrenal glands were removed. The adult rats were group-housed with three animals per cage with free access to food and water. The pups were used prior to weaning and therefore were housed with the dam. The humidity- and temperature-controlled vivarium was maintained on a 12-h light/dark cycle (lights on 7 am). All animal procedures were carried out in accordance with Home Office regulations and local ethics committee guidelines.

### Drug Preparation and Treatments

A 0.1 M (30 mg/ml) solution of all-*trans* RA in dimethyl sulfoxide (DMSO) was prepared and stored in the dark at −70°C until required. On the day of use, 1 ml of 0.1M RA was diluted in 29 ml of Dulbecco’s Modified Eagle’s Medium (DMEM) containing 10% fetal calf serum (FCS) to make a 1 mg/ml RA solution (Luo et al., 2004). The solution was stored in the dark until administration. The vehicle-only control solution consisted of 1 ml DMSO diluted in 29 ml DMEM + 10% FCS.

### *In vivo* Studies

RA was administered *via* an intraperitoneal route and 8-weeks old rats were given either 1 mg/kg, 10 mg/kg RA or an equivalent volume of DMSO-only control (*n* = 5 rats per treatment group). Rats were given either a single injection of RA/vehicle, or an injection once per day for three consecutive days. Injections were carried out in the morning and rats were then sacrificed 5 h after the final injection. Rats were euthanized by CO_2_ inhalation. Euthanasia was followed by cervical dislocation and the hippocampus, hypothalamus, pituitary and one of the adrenal glands were removed and stored at −70°C until processed.

### *Ex vivo* Studies

Rats were killed with Euthatal and a single adrenal gland from each rat was harvested immediately upon death. The adrenal gland was stored in a dish with culture medium (DMEM/F12, Gibco) on ice and taken for detailed dissection within approximately 1 h. The surrounding adipose tissue was removed from the adrenal gland under the microscope and each adrenal was cut in half to provide a within-animal control set. Each half-adrenal was transferred into a 24-well plate in 1 ml of culture medium (DMEM/F12 plus 10% FCS) and placed at 37°C, 5% CO_2_ for 2 h.

The adrenals were then treated with 1 μM RA or an equivalent concentration of DMSO (0.1%) for 5 h. Following treatment, the adrenals were removed from the wells and stored individually at −70°C before analysis.

### Quantitative PCR

Total RNA was extracted using an RNeasy mini kit (Qiagen) with on-column RNase-Free DNase treatment (Qiagen). Complementary DNA (cDNA) was synthesized from 500 ng total RNA using a qScript™ cDNA Synthesis Kit (CAT# 95047-100, Quanta Biosciences™). As yields from the cultured adrenal tissue were low, RNA was concentrated by ethanol precipitation before cDNA synthesis. Quantitative real-time PCR (qRT-PCR) was performed using the SensiMix SYBR master mix (Bioline) on a Roche LightCycler 480. The primers used for qPCR are listed in Table 1. Primers were designed using Primer-BLAST^1^ and synthesized by PrimerDesign Ltd or Sigma-Aldrich. *Actb* was used as the reference gene for all samples. All samples were run in triplicate, along with standard curves prepared from a 5-fold dilution of the stock cDNA samples mixed together and blanks. qPCR analysis was performed using relative quantification on Roche LightCycler 480 Software 1.5.1.

**Table 1:** Genes and primer sequences for qPCR.

Gene	Product size (bp)	Primer sequence (5′-3′)
*Rarb*	134	F-ACACCACGAATTCCAGCGCTGAC
		R- CAGACCTGTGAAGCCCGGCA
*Actb*	112	F- CCACACCCGCCACCAGTTCG
		R- TACAGCCCGGGGAGCATCGT
*Nr3c2*	83	F- CGTGCGGCTTCAGCTGACCT
		R- TGAGGCCATCTTTTGGAACTGTGCTC
*Fkbp5*	150	F- CGTGTGCCAGAGGAAGGCGA
		R- TGTTTCTTGCCGGCCGCTCC
*Hsd11b1*	121	F- TCACTGGGGCCAGCAAAGGGAT
		R- AGGCAGCGAGACACCACCTTCTG
*Crh*	132	F- CTGCCAAGGGAGGAGAAGAGAGCG
		R- TCCAGAGACGGATCCCCTGCTCA
*Nr3c1*	150	F- GCTGGAGGTGATTGAACCCGAGG
		R- CTGAAGCCTGGTATCGCCTTTGCC
*Pomc*	216	F- GCCTTTCCGCGACAGGGGTC
		R- AAGCCCGGATGCAAGCCAGC
*Pcsk1*	100	F- GTGCAAGCTGGGGCCCTAATGA
		R- CCTTGTCTCCCCTGTTTGACACCG
*Pcsk2*	137	F- GAATCCCGAGGCCGGTGTGG
		R- GCCAGGTCAGATCCACGTTAGCC
*Cyp11b1*	145	F-GGAGTGTCATATCCGAGATGGTAGCAC
		R- TCTGGGTTCCGAGCCAGCTCAA
*Cyp11b2*	122	F- TGGGTGGCCCACAGGGAACTC
		R- TTTTGAACAGCTTTTGGTGACAGCACG

### SDS-PAGE and Western Blotting Analysis

Tissues were homogenized in a buffer consisting of 150 mM NaCl, 1% Triton, 0.1% SDS, 50 mM HEPES and protease inhibitors (Roche). Protein concentrations were measured by BCA assay (ThermoFisher Scientific, Waltham, MA, USA). 50μg of protein was loaded and separated on 12% SDS-polyacrylamide gels and then transferred onto Hybond-ECL nitrocellulose membranes (Amersham Protran Premium 0.45 μm NC) using a Mini Trans-Blot Cell (Bio-Rad). The membranes were blocked with 5% skimmed milk in Tris-buffered saline containing 0.05% Tween-20 (TBST) for 1 h at room temperature. The blots were incubated overnight in primary antibody, against either CYP11B1 (1:300; MAB6020, Millipore) or CYP11B2 (1:1,000; anti-C11B2 antibody, Abcam) diluted in TBST containing 2% BSA at 4°C. The following day, the blots were washed three times with TBST before incubating with horseradish peroxidase-conjugated secondary antibody at 1:3,000 dilution in TBST containing 5% skimmed milk, using anti-mouse secondary antibody (Millipore) for CYP11B1 or anti-rabbit secondary antibody (Jackson ImmunoResearch Laboratories, Inc., West Grove, PA, USA) for CYP11B2, each incubated for 1 h at room temperature. The membranes were then washed three times for 5 min in TBST and incubated at room temperature for 2 h with HRP-conjugated anti-β-actin (1:50,000, Sigma). After three washes in TBST for 5 min, the blots were then developed using enhanced chemiluminescence substrate (Millipore) and the protein bands for CYP11B1 or CYP11B2 together with β-actin were imaged simultaneously using a MyECL Imager (Thermo Scientific Fisher, Waltham, MA, USA). Band intensities were quantified from images using ImageJ software 1.47v.

### Enzyme-Linked Immunosorbent Assay (ELISA)

Corticosterone and aldosterone levels in serum were measured by ELISA (CAT# KGE009 and KGE016, R&D Systems Biotechne for corticosterone and aldosterone, respectively). Serum samples were stored at −70°C, thawed and 150 μl of blood serum treated with 150 μl of pretreatment reagent supplied with the kit, incubated at room temperature for 15 min and subsequently centrifuged at 12,000 *g* for 4 min. All other steps were performed according to the manufacturer’s protocol and the microplate was read at 450 nm and 560 nm on a Precision Microplate Reader (Molecular Devices, San Jose, CA, USA) using the SoftMax Pro v 5.2 software.

### Statistical Analysis

All data are expressed as Mean ± standard error of the mean (SEM). Statistical analyses were performed using the IBM SPSS Statistic v 25 program and graphical presentation of data used Microsoft Office Excel 2018. All groups of samples were subjected to normal distribution test using the Kolmogorov–Smirnov test and samples set at 100%.

For the 5-h *ex vivo* experiment, the cultured adrenals and the 3 days *in vivo* experiment were analyzed using a two-tailed Student’s *t*-test. However, the 3 days dose concentration experiment were analyzed using a one-way analysis of variance (ANOVA) with Dunnett’s multiple comparison test.

The degree of significance value (*p*-value) less than *p* < 0.05 was considered statistically significant. A *p*-value less than 0.05 was designated with one asterisk (*), *p* ≤ 0.01 designated with two asterisks (**), *p* ≤ 0.001 designated with three (***) and *p* ≤ 0.0001 designated with four (****).

## Results

Given the known response of the HPA axis to chronic (6 weeks to 3 months) treatment with RA (Cai et al., 2010; Vilar et al., 2016), this study investigated whether a short duration (3 days) of RA treatment (10 mg/kg) may bring about relatively rapid changes in the regulatory components of the HPA axis. This amount of RA was chosen to be equivalent to the high dose treatment used therapeutically for acne (Cyrulnik et al., 2012) which is a mean dose of 1.6 mg/kg per day, taking into account allometric scaling for conversion between species based on body surface area (Nair and Jacob, 2016). This was compared to a low dose of 1 mg/kg of RA. In addition to the HPA axis, the effect of RA on the hippocampus was assessed as it may regulate HPA axis activity via feedback on to the hypothalamus (Jacobson and Sapolsky, 1991).

**Figure 1:**
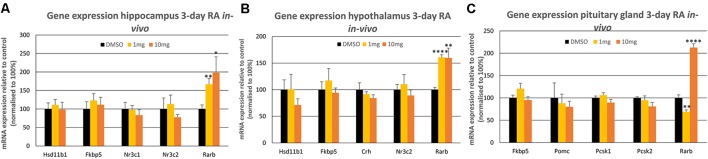
The influence of 3-day 1 mg/kg or 10 mg/kg retinoic acid (RA) treatment of the rat on mRNA expression levels in the **(A)** hippocampus, **(B)** hypothalamus and **(C)** pituitary gland. The *Rarb* positive control was the only gene to show a statistically significant change in the three tissues (*p* < 0.001 in all cases) indicated by *. All control values were normalized to 100%. Panel **A**; *n* = 6 RA, *n* = 6 control; panel **B**; *n* = 6 RA, *n* = 6 control and panel **C**; *n* = 5 RA, *n* = 5 control. * = *p* < 0.05 , ** = *p* < 0.01, **** = *p* < 0.0001.

In the hippocampus, the two receptors crucial for glucocorticoid negative feedback were investigated; the mineralocorticoid receptor (MR, encoded by *Nr3c2*) and the glucocorticoid receptor (GR, *Nr3c1*; McEwen et al., 1992). In addition, the gene for the GR co-chaperone FK506 binding protein 51 (*Fkbp5*; Hähle et al., 2019) which has been implicated in modulating GR sensitivity, was measured together with 11β-hydroxysteroid dehydrogenase type 1 (*Hsd11b1*, Wyrwoll et al., 2011) encoding an enzyme converting cortisone to active corticosterone. However, none of these genes, key to the modulation of glucocorticoid signaling, were influenced by short-term activation of RA signaling (Figure 1A). In contrast, the dose-dependent positive induction of RA receptor beta gene (*Rarb*), known to be highly responsive to RA (Sucov et al., 1990), demonstrated that RA reaches the hippocampus and that the cellular machinery required for a transcriptional response is present (Figure 1A).

A similar set of glucocorticoid regulatory genes were investigated in the hypothalamus following the same short-term treatment with RA, examining *Nr3c2*, *Fkbp5*, *Hsd11b1* as well as the gene encoding corticotropin-releasing hormone (*Crh*; Figure 1B), produced by the hypothalamus to induce ACTH release by the pituitary gland. Again, however, RA did not influence the expression of these genes in the hypothalamus, whereas the *Rarb* positive control was induced equally by 1 and 10 mg/kg RA. Finally, in the brain the influence of such RA treatment was investigated on the pituitary gland, determining the expression of *Nr3c2*, *Fkbp5* as well as *proopiomelanocortin* (*Pomc*), encoding the precursor of ACTH and the genes encoding the enzymes that cleave POMC, prohormone convertase 1 (PC1 protein, *Pcsk1* gene) and 2 (PC2 protein, *Pcsk2* gene; Figure 1C; Harno et al., 2018). As in the hippocampus and the hypothalamus, there were no significant changes in these genes in RA-treated rats, in contrast to the significant induction of the *Rarb* positive control by 10 mg/kg RA, however a downregulation of *Rarb* was evident at 1 mg/kg RA (Figure 1C).

**Figure 2:**
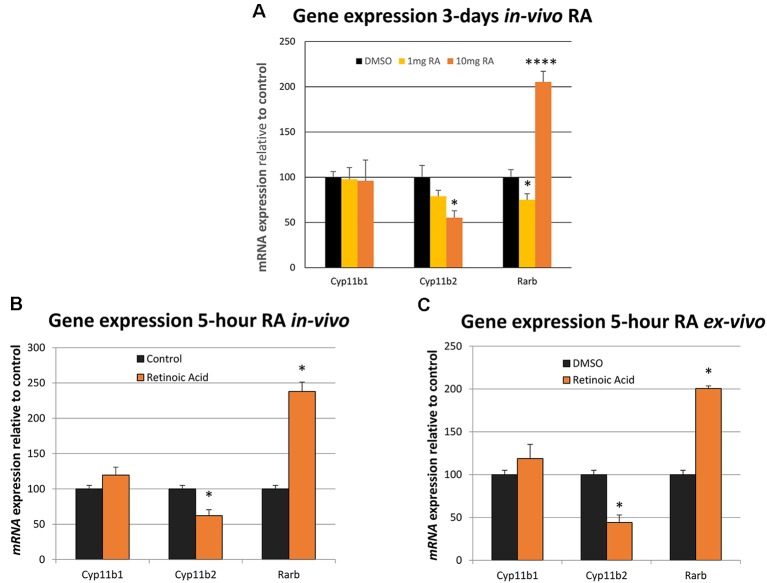
The influence of *in vivo* and *ex vivo* RA treatment on adrenal gland *Cyp11b1, Cyp11b2* and *Rarb* expression. **(A)** The relative effects of 1 mg/kg vs. 10 mg/kg dose of RA in the rat was compared on gene expression and there was no influence on *Cyp11b1* at either dose (1 mg/kg RA, *p* = 0.882, 10 mg/kg RA, *p* = 0.874) while *Cyp11b2* was repressed at the higher dose (1 mg/kg RA, *p* = 0.188, 10 mg/kg RA, *p* = 0.018). *Rarb* was strongly induced by the 10 mg/kg RA dose (*p* = 0.00009) but surprisingly repressed at 1 mg/kg RA (*p* = 0.048). **(B)** Five-hour treatment of the rat with RA resulted in significant induction of the *Rarb* positive control in the adrenal gland and *Cy11b2* fell in expression (*p* < 0.001 and *p* = 0.018, respectively). **(C)** Five-hour treatment with RA of an *ex vivo* adrenal glands culture resulted in *Rarb* again increasing and *Cyp11b2* falling in expression (*p* = 0.0188 and *p* = 0.0184, respectively). * = *p* < 0.05 , **** = *p* < 0.0001.

The influence of short-term RA treatment on gene expression was then investigated in the adrenal gland, the final component of the HPA axis, quantifying genes required for the synthesis of stress-regulating hormones. mRNA levels of the genes encoding the enzymes that catalyze the final rate-limiting step in corticosterone synthesis (*Cyp11b1*) and the synthesis of aldosterone from the same substrate (*Cyp11b2*; Schiffer et al., 2015) were measured by qPCR. 10 mg/kg RA induced a strong and significant repression of *Cyp11b2* and the *Rarb* positive control was induced by the same dose although again a downregulation of *Rarb* was evident at 1 mg/kg RA (Figure 2A). To determine whether RA had a rapid effect on these genes rats were treated with 10 mg/kg RA for just 5 h and this resulted in a significant reduction of *Cyp11b2* (Figure 2B). To determine if this influence was direct, independent of factors outside the adrenal gland, the tissue was removed, cultured *ex vivo* and the influence of RA studied after just 5 h (Figure 2C). *Cyp11b2* was again significantly repressed by RA treatment implying a direct rapid effect of RA on this gene, as is known to occur for RA induction of *Rarb* (Figure 2C).

**Figure 3:**
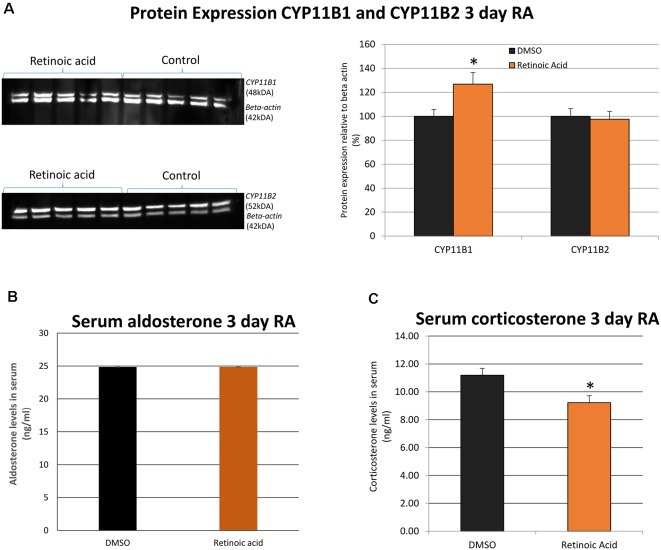
The influence of *in vivo* RA treatment on adrenal gland expression of CYP11B1 and CYP11B2 protein as well as serum aldosterone and corticosterone levels. **(A)** Western blotting and band quantitation to determine the change in CYP11B1 and CYP11B2 relative to β-actin (imaged simultaneously) following *in vivo* RA treatment showed a significant increase in CYP11B1 (*p* = 0.043) and no significant changes in CYP11B2 (*p* = 0.798). All control values were normalized to 100%. **(B)** Quantitation of blood aldosterone levels with *in vivo* RA treatment indicated no significant change (*p* = 0.221) while **(C)** quantitation of blood corticosterone levels indicated a significant decline in hormone after *in vivo* RA treatment (*p* = 0.049). Significance was indicated by *. N = 5 per treatment group in each case. *p < 0.05.

Protein expression of CYP11B1 and CYP11B2 was then examined after 3 days of RA treatment and CYP11B1 significantly increased with the application of RA (Figure 3A). In contrast, there was no significant change in CYP11B2 protein. Finally, plasma aldosterone and corticosterone were measured by ELISA to determine whether changes in these glucocorticoid-synthesizing enzymes in the adrenal gland translated into changes in blood hormone levels. The unexpected result was the lack of change in aldosterone and a decrease in corticosterone levels (Figures 3B,C) after RA treatment implying the action of RA on other mechanisms of control in addition to the changes in these synthetic enzymes.

## Discussion

The long-term influence of RA on the HPA axis has previously been examined from two perspectives; one as a treatment of Cushing’s disease (Vilar et al., 2016), and second as a possible contributor to depression (Cai et al., 2010, 2015; Hu et al., 2013). In the former, RA treatment over several months suppresses the HPA axis due to decreased ACTH secretion as a result of inhibition of pituitary gland POMC expression. The latter showed that approximately 3-weeks of RA exposure induces HPA activity, with increased CRH in the hypothalamic paraventricular nucleus (PVN) and decreased GR protein levels in the hypothalamus (Hu et al., 2013; Cai et al., 2015).

The present study contrasted these long-term actions with the short-term (3-day) effects of RA in the adult rat to indicate what may be the initial targets of RA in the HPA axis. The results paint another picture again of the action of RA on this axis with an apparent direct action on the adrenal gland. It was shown that RA reached all tissues of the HPA axis as demonstrated by induction of the RA-responsive gene *Rarb*. Many key genes involved in the HPA axis did not change with this treatment including those encoding the corticosteroid hormone receptors, CRH, as well as POMC and the enzymes processing POMC protein together with the 11β-HSD1 enzyme. The only early significant effect in the components of the HPA system examined in the adult rat was in the adrenal gland in which the *Cyp11b2* gene encoding the enzyme that takes the corticosterone substrates to aldosterone was rapidly down-regulated. That this is a direct effect is implied by the rapid (5 h) effect of RA when treated *in vivo* and that RA can directly induce this when applied to cultured rat adrenal gland. At the 3-day time point examined however there was no corresponding decrease in CYP11B2 protein. In contrast, there was a significant increase in the CYP11B1 protein which catalyzes the synthesis of corticosterone. Surprisingly, when the hormone product of this enzyme, corticosterone, was measured in the circulation, the levels did not reflect the increase in the synthetic enzyme, rather there was a decline while the hormone product of CYP11B2, aldosterone, remained unchanged.

Thus, RA treatment brings about three significant changes in the adrenal gland related to corticosterone synthesis in levels of transcript, protein and corticosterone itself, but the changes are surprisingly disconnected. Although RA appears to have a rapid influence on *Cyp11b2* expression this is not translated into a change in protein by 3 days. There are presumably compensatory mechanisms preventing protein response and although it is possible that protein may be decreased in the first day or two this would be unlikely to bring significant change to the adrenal gland. Intriguingly, the change in protein induced by RA was in a gene that did not change in transcript levels and this may indicate a direct effect of RA to regulate protein translation. This function of RA, independent of gene transcription, is amongst its repertoire of “non-genomic actions.” These actions include regulation of mRNA translation *via* direct interaction of RARα with transcript and binding of RA releasing mRNA to allow its translation (Maghsoodi et al., 2008; Schwertz et al., 2017). It may be conjectured that RA can regulate CYP26B1 through such a mechanism. It is unknown whether the level of change is sufficient to increase corticosterone synthesis and when serum levels were measured a contrary decline in corticosterone was evident. It is not immediately apparent how these shifts are integrated but they may represent a view at a single time point of 3 days of rapid fluctuations followed by feedback repression, for instance, the decline in corticosterone at 3 days may be a feedback response to an earlier increase. Such a rapid negative feedback system is well-characterized in the HPA axis (Tasker and Herman, 2011). This feedback suppresses the secretion of CRH from neurons within the PVN of the hypothalamus. There are a series of overlapping systems triggering this feedback overlapping in time intervals with the fastest taking seconds to minutes and the rapid actions of glucocorticoids are mediated *via* non-genomic actions triggering various steps including a G-protein coupled receptor rather than the slower transcriptional regulatory route using the nuclear GR (Di et al., 2003). Such non-genomic actions of GR are also proposed for the distinct rapid negative feedback action glucocorticoids have on pituitary production of ACTH (Deng et al., 2015). Such negative feedback is crucial to prevent hormone depletion and is also protective of harm from excess glucocorticoid exposure (Strelzyk et al., 2012; Johnstone et al., 2019). A decline in this feedback results in HPA hyperactivity and is a biological change frequently linked with depression (Pariante and Lightman, 2008). The multiple layers of feedback act to maintain correct levels of corticosteroids under a wide range of physiological conditions. These can be aberrantly disrupted though as a result of drug treatment. The action of RA on the adrenal gland is rapid and for instance, leads to a decrease in *Cyp11b2* transcript within 5 h. It will be presumed that any resulting change in corticosterone will happen in less than 24 h and the early changes as a result of RA treatment are expected to be an increase in corticosterone. Rapid feedback inhibition is known to be a response to increasing corticosterone concentrations, rather than absolute concentration (Abe and Critchlow, 1977). It is possible the result seen in this study at the 3-day period is the outcome of negative feedback and a slight overcompensation leading to a decline in corticosterone compared to controls. This can be resolved with future studies refining the analysis of these early changes and quantifying corticosterone in intervals up to 3 days. Parallels may also exist in these responses with the changes seen in *Rarb* expression, which generally responds with induction of expression but decreases with 1 mg/kg RA treatment in the pituitary and adrenal glands. This may be conjectured to be the result of feedback decline at this particular dose perhaps similar to the feedback response in RA signaling that can occur in the embryo (Lee et al., 2012).

The complex action of RA on the HPA axis is evident from long-term studies. Nineteen days of intracerebroventricular injection of RA into the rat increases CRH and RARα in the hypothalamus, as well as repressing GR and inducing a rise in blood corticosterone (Hu et al., 2013). A further view into the complication of the interactions of RA with the HPA axis is evident if RA levels are dropped in the rat as a result of 10-week deficiency of substrate (vitamin A). In this case, blood corticosterone rises, and 5-days of 0.15 mg/kg RA decreases this back to control levels. With such treatment, there are no changes in expression in the genes for *Nr3c2*, *Nr3c1* or *Crh* in the hypothalamus, although *Hsd11b1* rises when vitamin A is depleted and falls back to control levels with RA treatment. There are however changes in the hippocampus with vitamin A deficiency, inducing decline in the genes for MR and GR and decrease in *Hsd11b1*, all of which are restored by RA treatment (Marissal-Arvy et al., 2013).

The actions of RA on the adrenal gland may contribute to the range of side-effects found with RA when used as a drug, evident from when it first entered the market in the early 80 s for the treatment of acne. Although the most common effects are not severe they are very wide-ranging including effects on skin, muscle, bone, liver and central nervous system (CNS; Khalil et al., 2017). The teratogenic effects of RA have a well-established mechanistic basis but these other effects on the adult body are not well understood in terms of mechanism. Action of RA on the HPA axis could result in change in corticosteroid balance and provide a mechanism for the diverse adverse effects on multiple organs. As discussed earlier, an adverse effect of RA on hormonal balance, including cortisol concentrations, has previously been shown (Karadag et al., 2015). However, all previous work has been on the long-term effects of RA and this study is the first to show a rapid effect on the adrenal gland which may be one of the primary factors to disrupt the entire HPA axis. If there were a long-term reset of feedback in the HPA axis this could contribute to an explanation for some of the lasting adverse effects. For instance, joint pain, bone disease, skin disorders and inflammatory bowel disease have been reported to continue even after discontinuation of therapy (Brzezinski et al., 2017), whereas the half-life of the drug is only 29 h (Nulman et al., 1998).

To conclude, a high dose of RA, equivalent to that used for high dose treatment of acne, does not affect transcript levels in the short-term of several key genes of the neural part of the HPA axis. However, a response is evident in the adrenal gland where RA regulates *Cyp11b2* transcription and CYP11B1 protein levels in the adult adrenal gland which were decreased and increased, respectively. The surprising net effect of these changes is a decrease in circulating corticosterone at day 3, potentially the result of dynamic changes in corticosterone over time with feedback compensation resulting in a decline below normal levels. The results point to the early events following RA treatment, inducing small independent changes in various aspects of adrenal gland corticosteroid synthesis that may underlie the long-term effects of either activation or repression of the HPA axis with RA treatment (Cai et al., 2010, 2015; Hu et al., 2013; Vilar et al., 2016) and place early events in the adrenal gland as a key to these changes.

## Ethics Statement

This study was carried out in accordance with the recommendations of the UK Home Office guidelines. The protocol was approved by the UK Home Office as part of a project license.

## Author Contributions

PI: designed and performed experiments and co-wrote the manuscript. EB, PS and SM: designed and performed experiments. PM: designed experiments and co-wrote the manuscript.

## Conflict of Interest

The authors declare that the research was conducted in the absence of any commercial or financial relationships that could be construed as a potential conflict of interest.

## Footnotes

^1^https://www.ncbi.nlm.nih.gov/tools/primer-blast/
